# Intra-Auditory Integration Improves Motor Performance and Synergy in an Accurate Multi-Finger Pressing Task

**DOI:** 10.3389/fnhum.2016.00260

**Published:** 2016-06-08

**Authors:** Kyung Koh, Hyun Joon Kwon, Yang Sun Park, Tim Kiemel, Ross H. Miller, Yoon Hyuk Kim, Joon-Ho Shin, Jae Kun Shim

**Affiliations:** ^1^Department of Kinesiology, University of MarylandCollege Park, MD, USA; ^2^Department of Physical Education, Hanyang UniversitySeoul, South Korea; ^3^Neuroscience and Cognitive Science Program, University of MarylandCollege Park, MD, USA; ^4^Applied Mathematics and Statistics, and Scientific Computation Program, University of MarylandCollege Park, MD, USA; ^5^Department of Mechanical Engineering, Kyung Hee UniversityYongin-Si, South Korea; ^6^Department of Stroke Rehabilitation, National Rehabilitation CenterSeoul, South Korea; ^7^Department of Bioengineering, University of MarylandCollege Park, MD, USA

**Keywords:** intra-auditory integration, multi-finger synergy, multi-finger pressing, motor performance, hierarchical variability

## Abstract

Humans detect changes in the air pressure and understand the surroundings through the auditory system. The sound humans perceive is composed of two distinct physical properties, frequency and intensity. However, our knowledge is limited how the brain perceives and combines these two properties simultaneously (i.e., intra-auditory integration), especially in relation to motor behaviors. Here, we investigated the effect of intra-auditory integration between the frequency and intensity components of auditory feedback on motor outputs in a constant finger-force production task. The hierarchical variability decomposition model previously developed was used to decompose motor performance into mathematically independent components each of which quantifies a distinct motor behavior such as consistency, repeatability, systematic error, within-trial synergy, or between-trial synergy. We hypothesized that feedback on two components of sound as a function of motor performance (frequency and intensity) would improve motor performance and multi-finger synergy compared to feedback on just one component (frequency or intensity). Subjects were instructed to match the reference force of 18 N with the sum of all finger forces (virtual finger or VF force) while listening to auditory feedback of their accuracy. Three experimental conditions were used: (i) condition F, where frequency changed; (ii) condition I, where intensity changed; (iii) condition FI, where both frequency and intensity changed. Motor performance was enhanced for the FI conditions as compared to either the F or I condition alone. The enhancement of motor performance was achieved mainly by the improved consistency and repeatability. However, the systematic error remained unchanged across conditions. Within- and between-trial synergies were also improved for the FI condition as compared to either the F or I condition alone. However, variability of individual finger forces for the FI condition was not significantly decreased as compared to I condition alone. This result indicates an improvement in motor performance is consistent with Bayesian estimation, and changes in multi-finger interaction mostly result in the enhanced motor performance. These findings provide evidence that the central nervous system can take advantage of the intra-auditory integration in a statistically optimal (Bayesian) fashion to enhance motor performance by improving multi-finger synergy.

## Introduction

For nearly every real-world case of motor behavior, the central nervous system (CNS) simultaneously receives information from a variety of sensory modalities, including visual, tactile, and auditory signals (Ernst and Banks, 2002; Stein and Stanford, 2008; Berniker and Kording, 2011), and is required to coordinate multiple effectors (e.g., motor units, muscles, joints, limbs) to effectively perform the movement (Babinski, 1899; Castiello, 1997; Alexandrov et al., 1998; Shim et al., 2003b). Many theories of motor control argue that integration of these multiple sensory modalities with the synergistic control of multiple effectors is critical for successful or optimal motor performance (Ernst and Banks, 2002; Berniker and Kording, 2011; Pantall et al., 2012; Scholz et al., 2012). In effectively integrating multi-sensory information and controlling multi-effector system, the CNS faces at least two challenges: it must estimate the state of the system with sensory information that is corrupted by noise and transmission delays (Körding and Wolpert, 2004; Faisal et al., 2008), and it must solve the motor redundancy problem when deciding how effectors are used from a theoretically infinite set of possibilities (Bernstein, 1967; Gel'Fand, 1971; Ting and Macpherson, 2005; Latash et al., 2007).

Concerning the first problem (state estimation), previous studies on inter-sensory integration (e.g., visual and auditory modalities) have suggested that the CNS combines multiple sensory modalities to produce minimum variance in the state estimate. Bayesian inference is a promising approach to interpret integration of multiple sensory modalities and to define the statistically optimal way to maximize accuracy of the state estimation (Ernst and Banks, 2002; Berniker and Kording, 2011). Relatedly, each individual sensory modality consists of multiple components that can be perceived distinctively by the CNS, such as the color and shape of an object in vision, or the frequency and intensity of a sound in audition (Helmholtz and Ellis, 1885; Bloj et al., 1999). However, our knowledge of how the CNS integrates these different components within a modality (i.e., *intra*-sensory integration) is very limited. For example, most previous studies on intra-sensory integration were on vision (Jacobs, 1999; Knill and Saunders, 2003; Hillis et al., 2004), with relatively little known about intra-sensory integration of auditory feedback. Sound is physically a pressure wave transmitted through the air, and humans can normally hear sound waves with frequencies up to ~10,000 Hz and intensities up to ~120 dB. These two physical quantities (frequency, intensity) are the most salient features of sound that contribute to its perception, and they appear to be perceived independently by the CNS (Helmholtz and Ellis, 1885; Zagorski, 1975). However, it remains unclear if and how the CNS integrates auditory frequency and intensity for state estimation.

Concerning the second problem (motor redundancy), the control of multiple motor effectors has been studied at the level of muscles (Babinski, 1899; Smith, 1993; Ting and McKay, 2007), joints (Castiello, 1997; van der Kamp and Steenbergen, 1999; Latash and Jaric, 2002), and body segments (Alexandrov et al., 1998; Kim et al., 2012), including fingers (Latash, 2000; Latash et al., 2002, 2007; Shim et al., 2003b, 2005b, 2008). From these studies, the notion of motor synergy has been developed to describe the interactions between multiple motor effectors for the successful completion of a given motor task (Latash et al., 2007). One of our previous simulation studies suggested a central back coupling hypothesis showing that it was theoretically possible to generate the synergistic actions between motor effectors in a feedforward system without an active involvement of sensory feedback (Latash et al., 2005). However, several previous studies suggested that sensory feedback plays an important role in synergistic interactions between motor effectors (Ranganathan and Newell, 2008; Shim et al., 2012; Koh et al., 2015). Ranganathan and Newell (2008) found that the removal of visual sensory feedback from combined visual and tactile feedbacks resulted in decreased synergistic actions between finger forces in both within-trial (online) and between-trial (offline) during multi-finger pressing. In addition, our recent work showed that the removal of tactile feedback in multi-finger pressing tasks resulted in decreases in multi-finger synergies for online control (Koh et al., 2015). These studies suggest that sensory feedback plays critical roles in multi-finger synergies and sensory integration may also systematically influence the synergistic interactions between motor effectors. Especially, the results of these studies imply that sensory integration may lead to improvements in multi-finger synergy (i.e., decrease covariance between finger forces).

Previous studies have shown that multi-finger actions are controlled in a hierarchical manner with at least two hierarchical levels: individual finger actions at the lower level, and virtual finer (VF: an imagined finger producing the same mechanical effect as all fingers together) actions through the combined mechanical outcomes of individual finer actions at the higher level (Arbib, 1985; MacKenzie and Iberall, 1994; Baud-Bovy and Soechting, 2001; Shim et al., 2005a,b), at the higher level. In our recent work, we developed hierarchical variability decomposition (HVD) model to quantify the hierarchical organization of multi-finger actions with the separate analysis of within-trial (online) and between-trial (offline) motor behaviors (Koh et al., 2015). The HVD model decomposes the variability in the motor system into mathematically independent components each of which quantifies a distinct motor behavior. In the HVD model, the systematic error, consistency, and repeatability are quantified at the higher level. In a constant force production task, the systematic error reflects the CNS's ability to estimate the target force, and consistency reflects the CNS's ability to perform the task on a moment-to-moment basis (i.e., online variability), while repeatability reflects the ability to reproduce the same task goal on trial-to-trial basis (i.e., offline variability). The analysis of online and offline variability were previously used to infer different aspects of the control mechanism in redundant motor systems (Scholz et al., 2003; Ranganathan and Newell, 2008; Koh et al., 2015). In the HVD model, both consistency and repeatability can be explained by the sum of individual finger force variances and the amount of error compensation (or amplification) among individual finger forces (i.e., motor synergy; Latash et al., 2005; Martin et al., 2009) at the lower level. Here, we tested how the intra-auditory integration affects these several aspects of multi-finger actions quantified through the HVD model.

In the present study, we therefore investigated the effect of intra-auditory integration between frequency and intensity of sound on motor performance and motor synergy during a constant multi-finger force production. We hypothesized that motor task errors would decrease when both frequency and intensity components are presented to subjects as compared to the conditions that present only one of these auditory components to them, which would support Bayesian integration between frequency and intensity of sound. We also hypothesized that intra-auditory integration would be associated with enhanced multi-finger synergy, which would indicate an evidenced role of intra-sensory integration in the interactions between redundant motor effectors in humans.

## Methods

### Participants

Ten right-handed male volunteers (age 24.2 ± 1 years) participated in the study. Participants were free of neurological, psychiatric, speech-language, and motor impairments. No participant had more than 1 year of musical training. Participants provided written informed consent. All procedures were approved by the University of Maryland College Park Institutional Review Board.

### Experimental setup

Finger pressing forces at the distal phalanges were collected using load cells (Nano 17, ATI Industrial Automation, Apex, NC, US) at a sampling frequency of 1000 Hz with data acquisition hardware (6024E, National Instruments Corporation, Austin, TX, US) using a custom LabVIEW program (LabVIEW 8.2, National Instruments Corporation, Austin, TX, US). The program interfaced with a function generator (Agilent 33522A, Keysight Technologies, Inc., Santa Rosa, CA, US) to register the individual finger forces and calculate the virtual finger (VF) force as the sum of individual finger forces. The program also generated auditory signals played through the left and right ears of headphones worn by the subjects (AE2, Bose Corporation, Framingham, MA, US).

In order to minimize distortion of sound due to headphone frequency response characteristics (Jackson and Vinegar, 1979), the auditory signal was calibrated to produce a constant intensity across all frequencies. Calibration was performed in a soundproof room by manipulating frequency from 20 to 10,000 Hz in 1 Hz increments and normalizing intensity at each increment.

### Procedures

**Figure 1:**
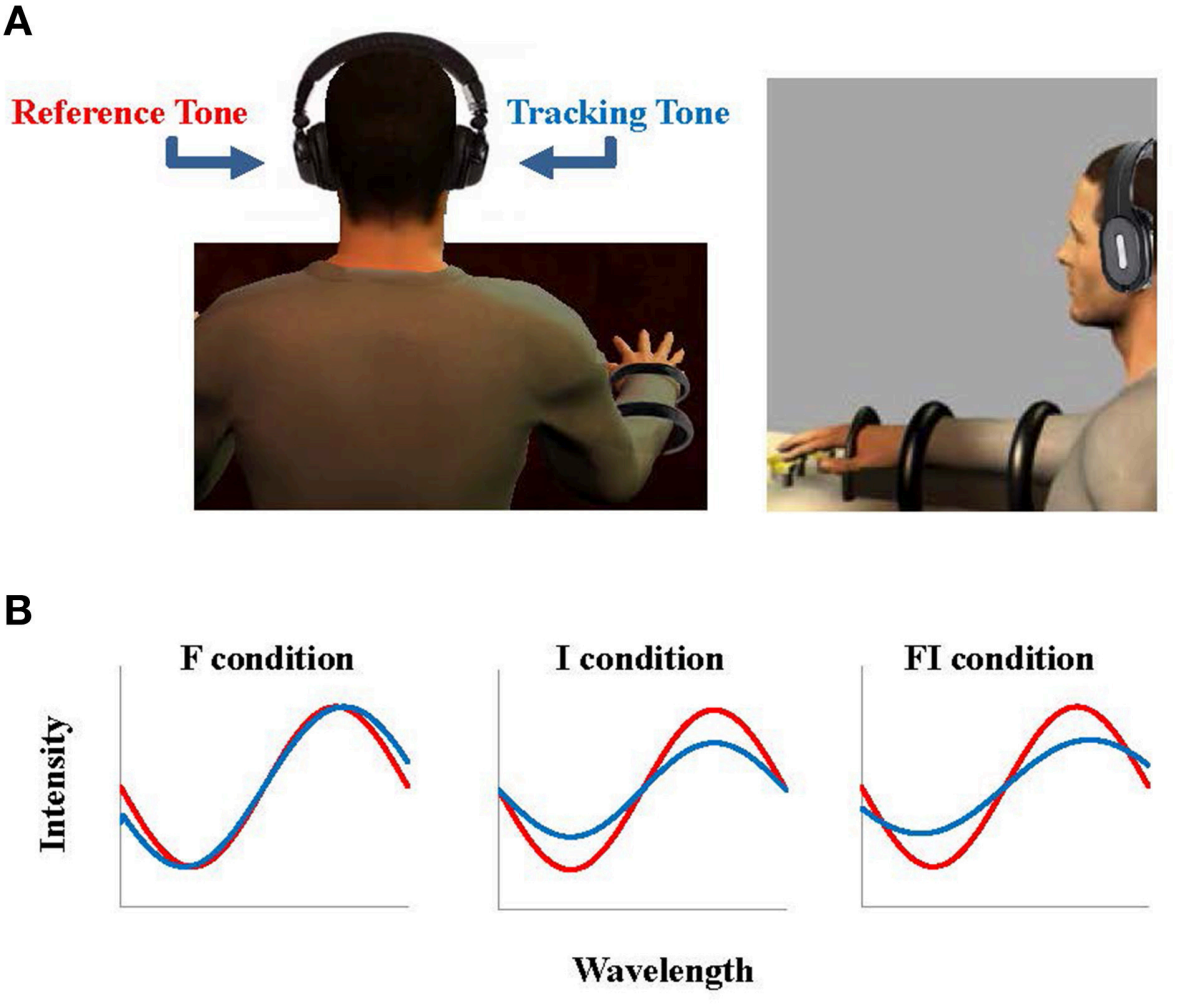
**Experimental setup**. The subjects sat and placed their right hand finger tips on the sensors, wearing the headphones **(A)**. During the task, the reference force (18 N) was provided as an auditory signal, a sinusoid signal with frequency 1000 Hz and intensity 70 dB (i.e., reference tone), played to the subject's left ear (**B** in red). The force generated by the subject was also provided as a sinusoid signal (i.e., tracking tone) to their right ear according to three feedback conditions: frequency (F) in which the frequency of the tracking tone changed, intensity (I) in which the intensity of the tracking tone changed, and frequency and intensity condition (FI) in which both the frequency and the intensity of the tracking tone changed according to the force the subject generated (**B** in blue).

Subjects sat on a chair, wore the headphones, and placed the tips of their right hand's fingers (index, middle, ring, and little) on the load cells (Figure 1). The subjects were asked to use these fingers to produce a constant VF force of 18 N over 20 s while they received auditory feedback tones on the reference force (left ear) and on the VF force (right ear). The tone for the reference force (i.e., the reference tone) had frequency of 1000 Hz and intensity of 70 dB. The tone for VF force (i.e., the tracking tone) varied in three different experimental conditions:

1. Frequency condition (F): the frequency of the tracking tone was modulated with the deviation of the subject's VF force from 18 N, while the intensity of the tracking tone was kept constant at 70 dB.
2. Intensity condition (I): the intensity of the tracking tone was modulated with the deviation of the subject's VF force from 18 N, while the frequency of the tracking tone was kept constant at 1000 Hz.
3. Frequency and Intensity condition (FI): both frequency and intensity of the tracking tone was modulated with the subject's VF force.

The frequency of 1000 Hz for the reference tone was used to minimize the influence of binaural beats (Oster, 1973; Wahbeh et al., 2007), and the intensity of 70 dB for the reference tone was selected as mid-level hearing. The feedback gains of frequency and intensity were 7 Hz/N and 0.7 dB/N, respectively, according to “just noticeable differences” of frequency and intensity previously reported assuming a change in force of 1 N (Moore, 1973; Ozimek and Zwislocki, 1996). Prior to the data collection, participants were given five familiarization trials that were excluded from analysis. From each 20-s trial, the 11-s window from 6 to 17 s where the VF force was relatively constant was extracted for analysis (Koh et al., 2015) in order to avoid the initial force stabilization in the beginning of each trial and premature cessation of force production at the end. Subjects completed 15 trials and with rest 30 s rest between trials to prevent fatigue. The order of conditions was balanced across the subjects.

### Analysis and bayesian model

The Bayesian approach in sensory integration research describes how sensory information can be used to update our knowledge of some quantity of interest (Jacobs, 1999; Ernst and Banks, 2002; Berniker and Kording, 2011). Our study had the following assumptions for analysis:

1. The Bayesian model for the sensory integration appropriate for static performance can also be used for dynamic performance (Ronsse et al., 2009). Let the random variable *S* be the true unknown VF force. If *S* has the specific value *s*, then (i) the estimated VF force based on frequency feedback, Ŝ_*F*_, and estimated VF force based on intensity feedback, Ŝ_*I*_, are independent Gaussian random variables with means *s*+*b*_*F*_ and *s*+*b*_*I*_ and variances $\sigma_{F}^{2}$ and $\sigma_{I}^{2}$, respectively, where *b*_*F*_ and *b*_*I*_ and are biases; (ii) the estimated VF force based on both frequency and intensity feedback, Ŝ_*FI*_, is the Bayesian estimate assuming a uniform prior over the range of feasible VF forces and ignoring the biases *b*_*F*_ and *b*_*I*_ (Scarfe and Hibbard, 2011; Shi et al., 2013). Let *s*+*b*_*FI*_ and $\sigma_{FI}^{2}$ denote the mean and variance, respectively, of the random variable Ŝ_*FI*_, where *b*_*FI*_ is the bias of the combined estimate.
2. The variances of VF force in the F, I, and FI conditions primarily reflect the estimation variances, $\sigma_{F}^{2}$, $\sigma_{I}^{2}$, and $\sigma_{FI}^{2}$, respectively. Additional variability in VF force caused by noise in other sensory systems and noise in the motor system is assumed to be small in all experimental conditions. The biases *b*_*F*_, *b*_*I*_, and *b*_*FI*_ equal *f*_*T*_ minus the mean VF force in the F, I, and FI conditions, respectively, where *f*_*T*_ is the reference force of 18 N.

Based on these assumptions of the Bayesian model, the posterior probability density describing our knowledge of *S* given specific measurements Ŝ_*F*_ = ŝ_*F*_ and Ŝ_*I*_ = ŝ_*I*_ is:

$$\begin{array}{l} p\left({Ŝ}_{FI}=s\right)=p\left(S=s|{Ŝ}_{F}={ŝ}_{F},{Ŝ}_{I}={ŝ}_{I}\right)\\ \;=\frac{p\left({Ŝ}_{F}={ŝ}_{F}|S=s\right)p\left({Ŝ}_{I}={ŝ}_{I}|S=s\right)}{\int_{-\infty }^{\infty }p\left({Ŝ}_{F}={ŝ}_{F}|S=s\right)p\left({Ŝ}_{I}={ŝ}_{I}|S=s\right)ds}. \end{array}$$

The posterior has a Gaussian distribution. Its mean, ŝ_*FI*_, is a weighted sum of the estimates ŝ_*F*_ and ŝ_*I*_:

$$\begin{array}{l} {ŝ}_{FI}={\text{w}}_{\text{F}}{ŝ}_{F}+{\text{w}}_{\text{I}}{ŝ}_{I}, \end{array}$$

where ${\text{w}}_{\text{F}}=\frac{\sigma_{I}^{2}}{\sigma_{F}^{2}+\sigma_{I}^{2}}$ and ${\text{w}}_{\text{I}}=\frac{\sigma_{F}^{2}}{\sigma_{F}^{2}+\sigma_{I}^{2}}$. The variance of the posterior, $\sigma_{FI}^{2}$, is smaller than the variances of the frequency estimate, $\sigma_{F}^{2}$, and intensity estimate, $\sigma_{I}^{2}$:

$$\begin{array}{l} \sigma_{FI}^{2}=\frac{\sigma_{F}^{2}\sigma_{I}^{2}}{\sigma_{F}^{2}+\sigma_{I}^{2}} \end{array}$$

The combined bias *b*_*FI*_ can also be expressed as a weighted average of the F condition bias, *b*_*F*_, and the I condition bias, *b*_*I*_ with the weights, w_F_ and w_I_ (Scarfe and Hibbard, 2011; Shi et al., 2013):

$$\begin{array}{l} b_{FI}={\text{w}}_{\text{F}}b_{F}+{\text{w}}_{\text{I}}b_{I} \end{array}$$

To test whether intra-auditory components, frequency and intensity, are integrated for the motor performance enhancement, we calculated the overall mean-squared error (*OMSE*) from the experimental data, the averaged squared deviation of the VF force from the reference force as a measure of motor performance:

$$\begin{array}{l} OMSE=\frac{1}{N}\overset{N}{\underset{i=1}{\sum }}\left\{\frac{1}{\tau }\int {\left[f_{T}-y_{i}\left(t\right)\right]}^{2}dt\right\} \end{array}$$

where *y*_*i*_(*t*) is VF force at trial *i*, and τ is the duration of *y*_*i*_(*t*).

In order to examine if the intra-auditory integration follows the Bayesian model, *OMSE*_*FI*_ was also estimated according to Bayesian model using the bias and variance values we experimentally obtained from the F condition and I condition:

$$\begin{array}{l} {OMSE}_{FI}=\sigma_{FI}^{2}+b_{FI}^{2}=\frac{\sigma_{F}^{2}\sigma_{I}^{2}}{\sigma_{F}^{2}+\sigma_{I}^{2}}+{\left({\text{w}}_{\text{F}}b_{F}+{\text{w}}_{\text{I}}b_{I}\right)}^{2} \end{array}$$

**Figure 2:**
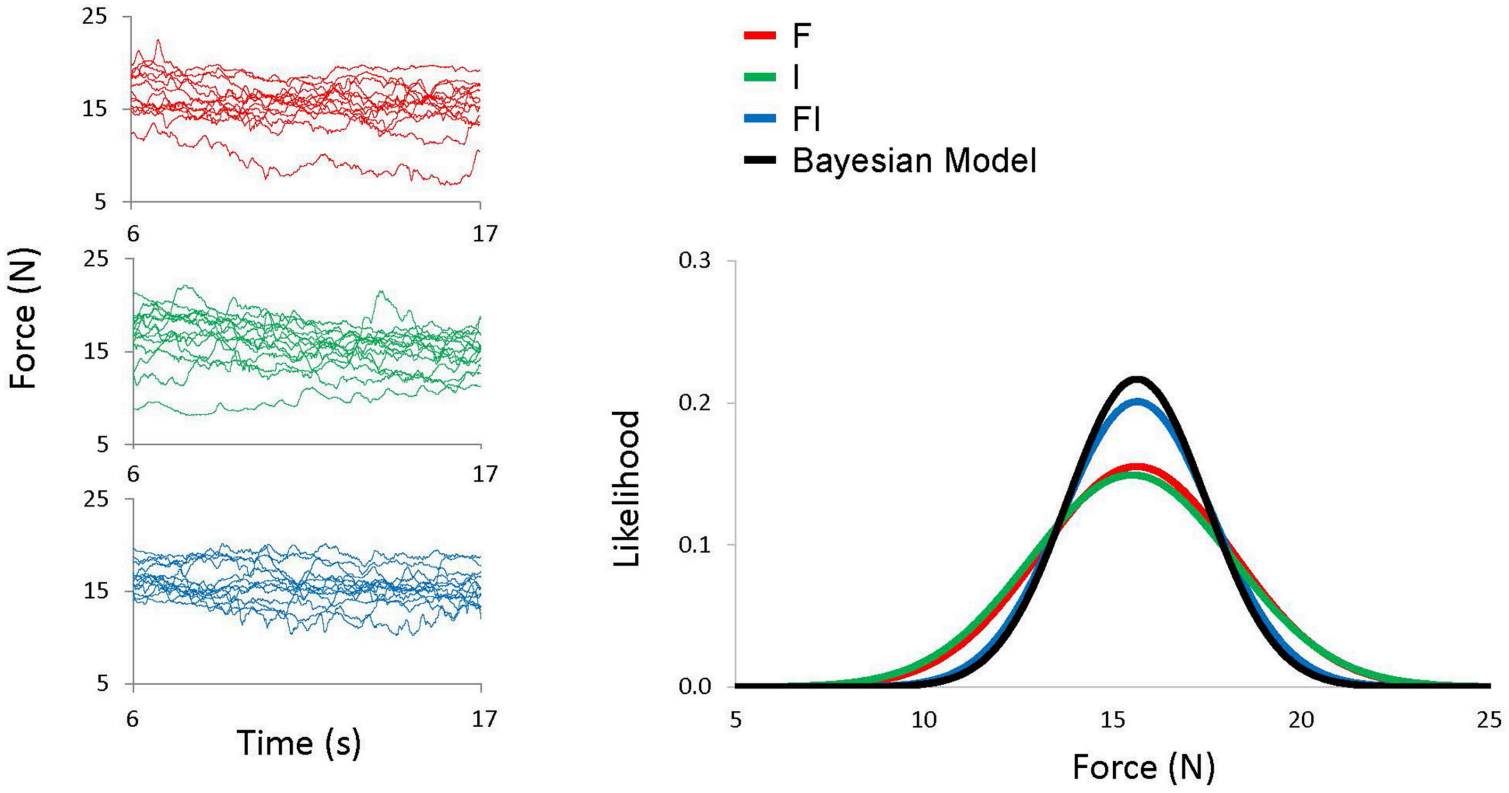
**Left figures show VF force time courses for all 15 trials from a representative subject for F condition (red), I condition (green), and FI condition (blue)**. Right Figure shows best fitted Gaussian distribution of VF force for all conditions and a Gaussian distribution calculated by the Bayesian model (black).

*OMSE*_*FI*_ estimated from the model was then compared to *OMSE* obtained from the experiment (Figure 2).

### Hierarchical structure and decomposition of motor variability

The VF force for trial *i*, *y*_*i*_(*t*), was modeled as the sum of three components:

$$\begin{array}{l} y_{i}\left(t\right)=X_{i}\left(t\right)+E_{i}+m \end{array}$$

where *X*_*i*_(*t*) is the demeaned VF force for trial *i*, *m* is the mean VF force after averaging over all time steps of all 15 trials, and *E*_*i*_ is the difference between the mean VF forces for trial *i* and *m*.

*OMSE* was partitioned into three error components that influence the motor performance following the HVD model (Koh et al., 2015): the “online variance” $\bar{\sigma_{X}^{2}}$, defined as the variance within a trial, averaged over trials, the “offline variance” $\sigma_{E}^{2}$, defined as the variance between trials, and the “systematic error,” *b*^2^, defined as squared deviation between the reference force and *m*. The sum of online and offline variances is the variance of the VF force ($\sigma^{2}=\bar{\sigma_{X}^{2}}+\sigma_{E}^{2}$), and the systematic error is the squared bias of the VF force ($b_{FI}^{2}={\left(18N-m\right)}^{2}$). The online and offline variances can also be defined in terms of the individual finger force variances and covariances as shown below.

$$\begin{array}{l} OMSE=\bar{\sigma_{X}^{2}}+\sigma_{E}^{2}+b^{2}=\bar{\overset{n}{\underset{i=1}{\sum }}\sigma_{x_{i}}^{2}}+\bar{\overset{n}{\underset{i\neq j}{\sum }}\sigma_{x_{i},x_{j}}}+\overset{n}{\underset{i=1}{\sum }}\sigma_{e_{i}}^{2}\\ \;+\overset{n}{\underset{i\neq j}{\sum }}\sigma_{e_{i},e_{j}}+b^{2} \end{array}$$

where *n* is the number of task fingers (*n* = 4), $\sigma_{x_{i}}^{2}$ and σ_*x*_*i*__, _*x*_*j*__ are a variance of within-trial *i*th finger force and a covariance between within-trial *i*th and *j*th finger forces, respectively, and $\sigma_{e_{i}}^{2}$ and σ_*e*_*i*__, _*e*_*j*__ are a variance and covariance for between-trial finger forces, respectively. The overhead bars indicate means over trials.

**Figure 3:**
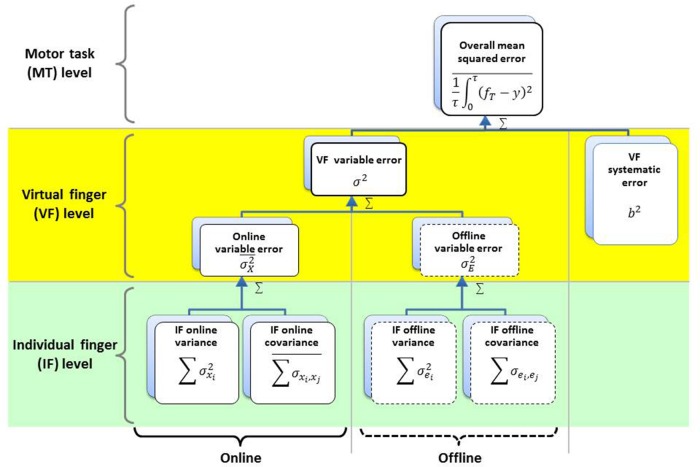
**Hierarchical organization of multi-finger force variability**. The overall mean squared error (OMSE) is composed of or the linear sum of the variance of the VF force (σ^2^) and the systematic error (*b*^2^). The variance of the VF force (σ^2^) is composed of or the linear sum of the intra-trial moment-to-moment variance (online variance; $\bar{\sigma_{X}^{2}}$), the time-averaged trial-to-trial variance (offline variance; $\bar{\sigma_{E}^{2}}$), and at the virtual finger (VF) level where the task is performed with the sum of all finger forces (VF force). The online and offline variances at the VF level are composed of or the linear sum of individual finger (IF) force variances $\left(\bar{\sum_{i\;=\;1}^{n}\sigma_{x_{i}}^{2}}\text{ and }\sum_{i=1}^{n}\sigma_{e_{i}}^{2}\right)$ and between-finger force covariances $\left(\bar{\sum_{i\neq j}^{n}\sigma_{x_{i},x_{j}}^{2}}\text{ and }\sum_{i\neq j}^{n}\sigma_{e_{i},e_{j}}\right)$ at the IF level.

According to the HVD model (Figure 3), the online and offline errors can be further defined as the sum of the individual finger force variances, plus between-finger covariances. Here, we use the covariances between finger forces, $\bar{\sum \sigma_{x_{i},x_{j}}}$ and ∑σ_*e*_*i*__, _*e*_*j*__, as indices of multi-finger synergy for online and offline, respectively (Koh et al., 2015). Negative covariance indicates that the individual finger forces synergistically act to compensate each other errors and attenuate the VF force error (i.e., error compensation), while positive covariance means that individual finger forces act synergistically, but each finger's errors are accumulated and the VF force error increases (i.e., error amplification; Shim et al., 2003a; Latash et al., 2007). The covariances between finger forces, $\bar{\sum \sigma_{x_{i},x_{j}}}$ and ∑σ_*e*_*i*__, _*e*_*j*__ that we use as an index of multi-finger synergy are mathematically similar to the motor synergy indices used in other previous studies (Shim et al., 2004; Latash, 2008, 2010; Ranganathan and Newell, 2008; Karol et al., 2011). One advantage of using covariances is that the total variance can be linearly decomposed into individual finger variances and inter-finger covariances (Koh et al., 2015). Therefore, covariances were used in this study as indices of motor synergies. We define motor synergy as task-specific interactions between motor effectors employed by CNS for enhancement of motor performance and utilization of degrees of freedom in a redundant motor system (Latash et al., 2007; Shim et al., 2008). The sum of individual finger force variances is the total variance in the motor task, while the between-finger covariance reflects synergistic interactions of individual finger forces. The total variance of the VF force (i.e., the sum of individual finger variances) was also decomposed into task-relevant and task-irrelevant variances according to the Uncontrolled Manifold theory (Scholz and Sch¨oner, 1999; Shim et al., 2004; Latash, 2010) to examine the task-relevant and task-irrelevant variances related to motor synergy.

### Statistical analysis

Paired *t*-test was used to compare *OMSE*_*FI*_ experimentally obtained and *OMSE* statistically estimated. One-way repeated-measures ANOVA with Fisher's *post hoc* pairwise multiple comparisons were used to compare three feedback conditions. A simple regression analysis was performed to determine whether either of mean or variance of VF force at each trial increase or decrease as a function of trial. The level of statistical significance was set at *p* = 0.05.

## Results

### Effects of intra-auditory integration on motor performance

**Figure 4:**
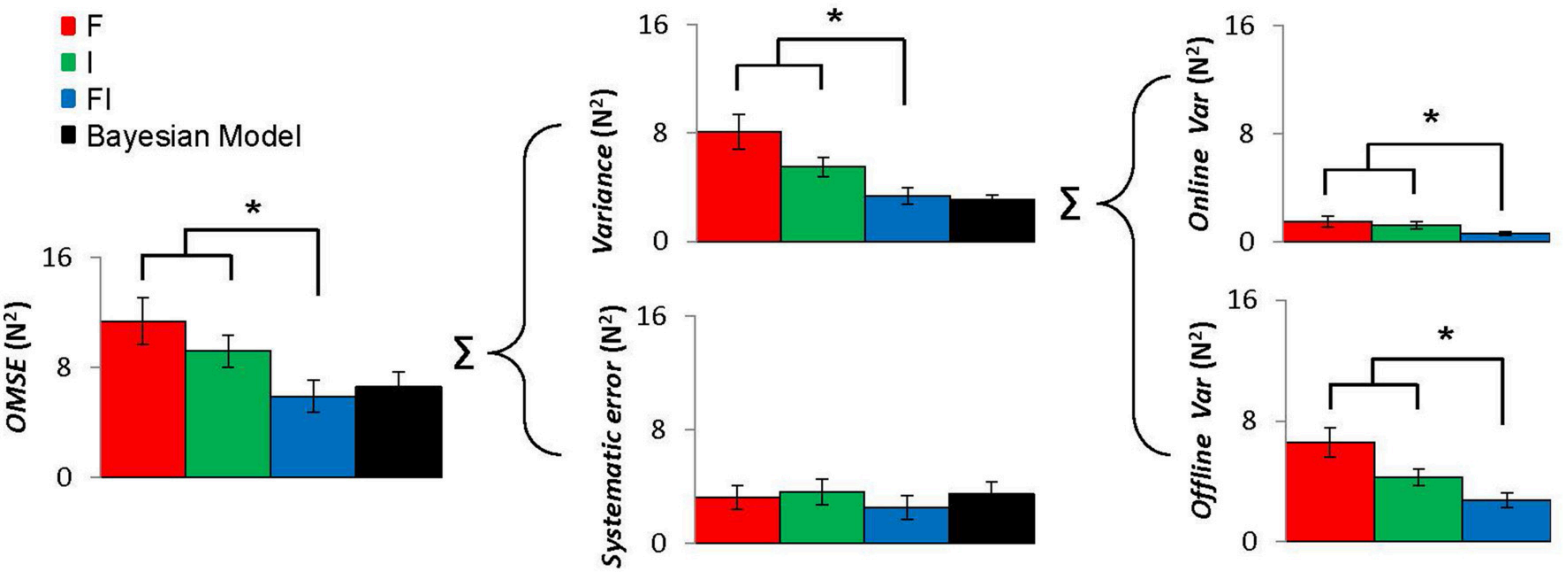
**VF level variables: overall mean square error (OMSE), varaince, sestematic error, and online and offline variances at the VF level for F condition (red), I condition (green), and FI condition (blue)**. These variables are associated with task performance. Error bars represent s.e.m. across subjects. OMSE calculated from the Bayesian model is also shown. Asterisk indicates statistical significance (*P* < 0.05).

We found that intra-auditory integration followed Bayesian model. The *OMSE*_*FI*_ calculated from the Bayesian model did not differ from the experimentally obtained *OMSE* [*t*_(9)_ = 0.896, *p* = 0.393]. There were also no differences between the model and the experimental data for VF variance [*t*_(9)_ = 0.614, *p* = 0.554] or the systematic error [*t*_(9)_ = 2.10, *p* = 0.065; Figure 4]. We also found that intra-auditory integration enhanced motor performance quantified as *OMSE*. Repeated-measures ANOVA showed that *OMSE* experimentally obtained from the FI condition was smaller than both *OMSE*_*F*_ and *OMSE*_*I*_ from the F condition and the I condition, respectively [*F*_(2, 9)_ = 12.76, *p* < 0.001; FI vs. F: *p* = 0.003, and FI vs. I: *p* = 0.004]. Using HDV model, *OMSE* decomposed into online and offline variance of the VF force, and the systematic error, which are reflected as consistency, reproducibility, and accuracy of motor task, respectively. We found that the enhanced motor performance achieved by improving consistency and reproducibility of the VF force with unchanged accuracy. The variance of the VF force in the FI condition was also smaller than those from the F condition and the I condition for both online control [*F*_(2, 9)_ = 8.92, *p* = 0.002; FI vs. F: *p* = 0.009, and FI vs. I: *p* = 0.002] and offline control [*F*_(2, 9)_ = 10.07, *p* = 0.001; FI vs. F: *p* = 0.003, and FI vs. I: *p* = 0.015]. The systematic errors did not differ across the feedback conditions [*F*_(2, 9)_ = 2.088, *p* = 0.153]. Thus, the decreases in *OMSE* were mainly due to the reduction of both online and offline variances, rather than systematic errors.

### Effects of intra-auditory integration on motor synergy

**Figure 5:**
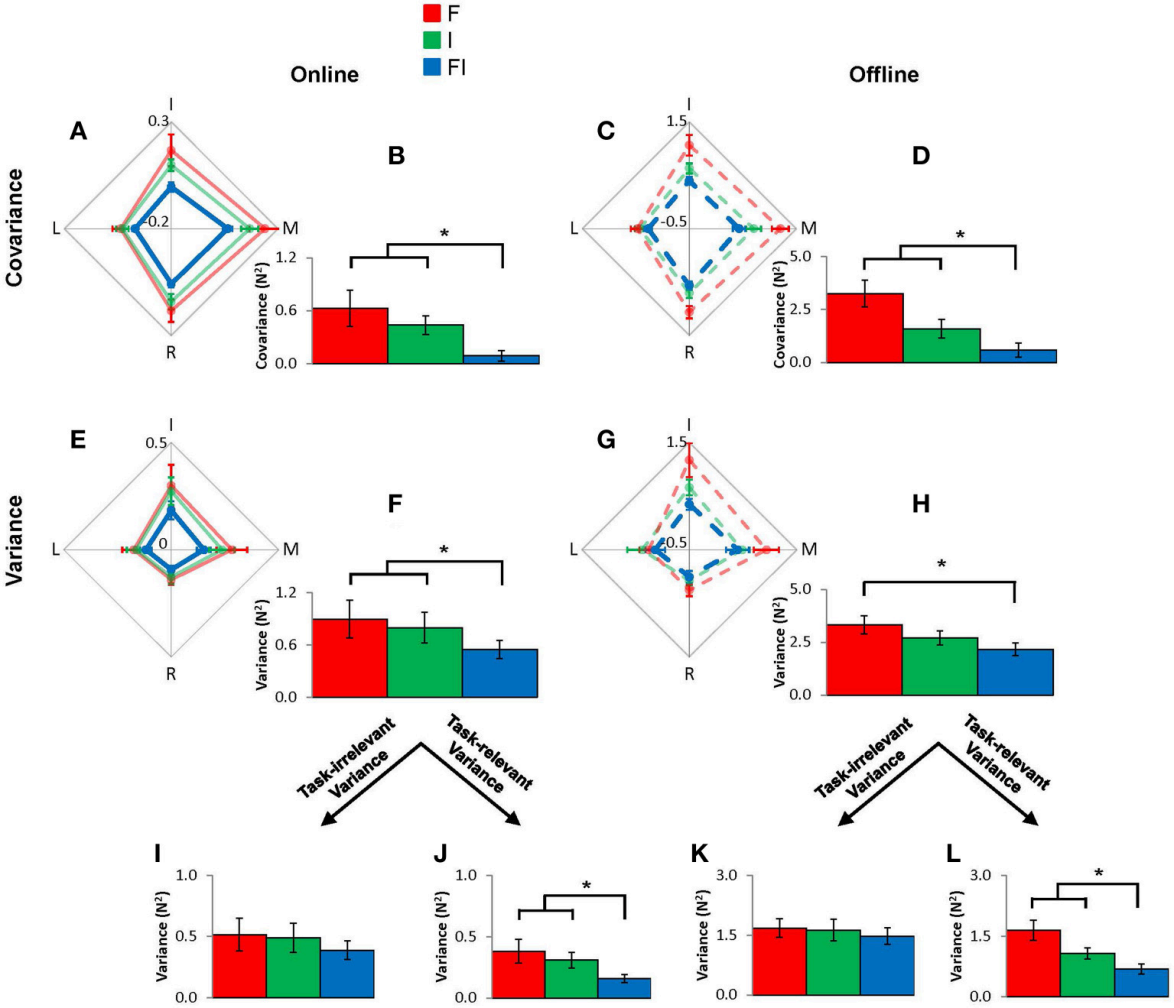
**Multi-finger force covariance and individual finger force variance for online and offline controls for F condition (red), I condition (green), and FI condition (blue), at the individual finger (IF) level**. The individual fingers' contributions to the overall covariance and variance values are shown in the 4-dimensional diamond graphs **(A,C,E,G)**. Error bars represent s.e.m. across subjects. The accompanying bar graphs show the overall covariance and variance values **(B,D,F,H)**. The covariance and variance, only in online control, showed statistically significant differences between FI and either F or I conditions. The overall variance values are decomposed into task-relevant and task-irrelevant variance values **(I–L)**. Task-relevant variance for both online and offline controls showed statistically significant differences between FI and either F or I condition. Asterisk indicates statistical significance (*p* < 0.05).

The VF variance in this model is mathematically equivalent to the sum of individual finger variances and covariances for both online and offline control, which allows us to investigate the source of changes in VF variances. We found that intra-auditory integration enhanced both online and offline motor synergy. Repeated-measures ANOVA revealed that online individual finger variance [*F*_(2, 9)_ = 6.86, *p* = 0.006; FI vs. F: *p* = 0.019, and FI vs. I: *p* = 0.016] and covariance [*F*_(2, 9)_ = 7.38, *p* = 0.005; FI vs. F: *p* = 0.011, and FI vs. I: *p* = 0.003] were lower in the FI condition than in the F and I conditions (Figure 5). In contrast, offline IF variance in the FI condition was smaller than the F condition, but there was no difference between the FI condition and the I condition [*F*_(2, 9)_ = 9.92, *p* = 0.001; FI vs. F; *p* = 0.002, and FI vs. I; *p* = 0.064]. Offline individual finger covariance was smaller in the FI condition than the F and I conditions [*F*_(1.276, 9)_ = 9.85, *p* = 0.006; FI vs. F: *p* = 0.004, and FI vs. I: *p* = 0.019]. Note that smaller individual finger covariance represents smaller error amplification between individual finger forces (Koh et al., 2015). In analyses of task-relevant and task-irrelevant spaces, both online and offline individual finger variances in the task-relevant space were smaller in the FI condition as compared to the F and I conditions [online: *F*_(2, 9)_ = 8.92, *p* = 0.002; FI vs. F: *p* = 0.009, and FI vs. I: *p* = 0.002, and offline: *F*_(2, 9)_ = 10.07, *p* = 0.001; FI vs. F: *p* = 0.003, and FI vs. I: *p* = 0.015], while both online and offline individual variances in task-irrelevant space remained unchanged throughout the feedback conditions [online: *F*_(2, 9)_ = 2.725, *p* = 0.093, and offline: *F*_(2, 9)_ = 1.001, *p* = 0.387]. Online individual finger covariance in both F and I conditions was statistically greater than zero [F: *t*_(9)_ = 3.020, *p* = 0.014 and I: *t*_(9)_ = 3.997, *p* = 0.003] while online covariance in FI condition did not significantly differ from zero [*t*_(9)_ = 1.429, *p* = 0.187]. Offline individual finger covariance in all conditions was statistically greater than zero [F: *t*_(9)_ = 6.056, *p* < 0.001, I: *t*_(9)_ = 6.845, *p* < 0.001, and FI: *t*_(9)_ = 4.326, *p* = 0.002].

### Learning effects

We also tested whether a learning effect exists using a simple linear regression. The mean and variance of VF force across time at each trial was calculated to test if these values significantly increase or decrease as a function of trial. No significant regression was found for mean of VF force in any of the conditions [F: *F*_(1, 13)_ = 4.296, *p* = 0.059, I: *F*_(1, 13)_ = 0.050, *p* = 0.827, and FI: *F*_(1, 13)_ = 0.108, *p* = 0.748], along with no significant regression for variance [F: *F*_(1, 13)_ = 4.515, *p* = 0.053, I: *F*_(1, 13)_ = 2.313, *p* = 0.152, and FI: *F*_(1, 13)_ = 2.392, *p* = 0.146].

## Discussion

Previous studies on sensory integration have mainly focused on the CNS mechanisms of *inter*-sensory integration, e.g., visual and auditory, visual and tactile, or visual and proprioceptive (Ernst and Banks, 2002; Alais and Burr, 2004; Helbig and Ernst, 2007; Butler et al., 2010; Fetsch et al., 2010; Reuschel et al., 2010). The current study, for the first time, showed how *intra*-sensory integration of frequency and intensity in auditory feedback affected motor performance and multi-finger synergy in a constant finger force production task. We reported that motor performance could be enhanced through intra-auditory integration as evidenced by improved tracking of the target force by the VF force. In addition, we report that multi-finger synergy in producing that VF force is enhanced through intra-auditory integration as evidenced by decreased covariance between individual finger forces.

Our study employed the hierarchical organization of multi-finger actions and used the HVD model previously proposed by our group for the separate analysis of within-trial (online) and between-trial (offline) motor behaviors (Koh et al., 2015). We found that providing feedback on both intensity and frequency was associated with the decreased covariances among individual finger forces in both online and offline control. This result indicates that the CNS responded to the presentation of feedback on multiple auditory components by decreasing error amplification among individual finger forces, resulting in both greater consistency and greater repeatability of the VF force.

Positive covariance between individual finger forces indicates that individual finger forces synergistically interact (Latash, 2010), but amplify the VF force error in the constant multi-finger pressing task, while negative covariance implies that individual finger forces interact to attenuate the VF force error, or to maintain a relatively constant VF force when individual finger forces are fluctuating. The ability to produce a particular VF with fluctuating individual finger forces can be viewed as a sign of versatility or flexibility within the motor system. The current study showed that the intra-auditory integration was associated with decreased positive covariance between individual finger forces, i.e., decreased error amplification at VF level. Many previous studies that employed visual force feedback often reported negative covariance between individual finger forces in similar tasks of constant VF force production (Shim et al., 2003b, 2004; Latash, 2010; Karol et al., 2011). Contrary to these studies, our study showed positive covariance between individual finger forces. This covariance difference may be due to the differences in the level of feedback uncertainty in sensory feedback the CNS received, leading to relatively high task uncertainty introduced by the auditory feedback used in our study as compared to the visual feedback used in the previous studies. This speculation is consistent with the study by Ranganathan and Newell (2008) in which they demonstrated that the covariances between individual finger forces could be systematically modulated by the uncertainty of the sensory feedback. Reduction in positive covariance between individual finger forces with intra-auditory integration would also be consistent with the principle of minimal interactions (Gelfand and Tsetlin, 1966), where interactions between low-level elements of a hierarchy are organized such that the inputs from one element to another (i.e., interactions) are minimized in an optimal system.

In addition, it was observed that offline individual finger variances and covariances were 2–5 times greater than online counterparts. The large differences between online and offline control suggest that the individual finger forces fluctuated more and the coupling between individual finger forces were stronger between trials as compared to within a trial. This observation indicates that the CNS used larger “workspaces” of individual finger forces and the inter-finger interactions were greater between individual finger forces in offline control than in online control. The large fluctuation of individual fingers forces in offline may be contributed by two different mechanisms: active utilization of the redundant degrees of freedom in the multi-finger system in offline control, and/or greater noise in the offline part of the control system. According to the Bayesian model, prior knowledge about the relative likelihood of different stimulus values can be integrated with new sensory information to improve estimates of stimulus values (Körding and Wolpert, 2004). Prior knowledge is task-specific and achieved through experience with that particular task. Although our analysis assumes no prior knowledge, it may be possible that prior knowledge gained from previous moments within a trial or previous completed trials may have contributed to the motor outcomes. If it is indeed the case, it is more likely that the prior knowledge influenced motor behaviors more within a trial as the sensory feedback is continuously provided online contrary to the offline counterpart. Future research specifically on prior knowledge and its impact on both online and offline control may enhance our understanding of the role of intra-auditory integration in motor performance and motor synergy.

Sensory integration research has often used congruent or incongruent multi-sensory information to provide sensory feedback for the same or a different state of physical property, respectively. For example, sound and light signals can come from a same position (congruency) or different positions (incongruency) in an experiment where a subject is instructed to identify the location of the sensory source (Battaglia et al., 2003). The previous studies showed that both congruent and incongruent feedback conditions follow the Bayesian model (Knill and Pouget, 2004; Körding and Wolpert, 2004). Our research investigated only the congruent auditory feedback where we provided the information about the same state of sound due to the technical difficulties to provide different reference force levels required for incongruent auditory feedback. According to the previous studies, force magnitude is associated with force variability (Schmidt et al., 1979; Galganski et al., 1993; Enoka et al., 1999). In other words, the force variability can affect motor performance and potentially motor synergy as well. Because of these reasons, our experiment did not involve incongruent auditory feedback in the effort to prevent adding additional complexity in the study design.

The feedback gains used in the current study were set according to “just noticeable differences” previously reported assuming a change in force of 1 N. The use of the feedback gains through individual auditory sensitivity test may have provided more accurate measures into the intra-auditory integration. In addition, we manipulated two physical quantities, frequency, and intensity. These quantities are transformed as physiological quantities, pitch, and loudness. The psychophysical transformation from frequency-intensity space to pitch-loudness space may not be a homeomorphism. However, the sensitivity test during a constant force production task demands a new method for accurate quantification through careful experiments and modeling because the just noticeable difference may be subject to changes during the dynamic process of finger force production.

In conclusion, we found that both motor performance and motor synergy during a constant multi-finger force production were enhanced through intra-auditory integration, which supports the idea that the CNS can integrate multiple sensory components within the auditory modality to enhance motor outputs consistent with the Bayesian model. In addition, our results provide new evidence that the intra-auditory integration is also associated with improved motor synergy. Although the central back coupling hypothesis proposed in our previous study demonstrated the theoretical possibility of generating synergistic actions between motor effectors without an active involvement of sensory feedback (Latash et al., 2005), the current study provides evidences that the auditory feedback and intra-auditory integration can indeed play an important role multi-finger synergy.

## Author contributions

Study conception and design: KK, HK, and JS. Acquisition of data: KK, HK. Analysis and interpretation of data: KK, HK, and JS. Drafting of manuscript: KK and JS. Critical revision: YP, TK, RM, YK, and JS.

### Conflict of interest statement

The authors declare that the research was conducted in the absence of any commercial or financial relationships that could be construed as a potential conflict of interest.
